# Testicular morphometry of rats with Walker 256 tumor supplemented with L-glutamine

**DOI:** 10.1590/1984-3143-AR2020-0051

**Published:** 2021-07-19

**Authors:** Nayara Rodrigues Rocha, Janine Karla França da Silva Braz, Sara Raquel Garcia de Souza, Luciane Fracaro, Fabiana Cristina Silveira Alves de Melo, Jacqueline Nelisis Zanoni, Naianne Kelly Clebis, Danielle Barbosa Morais, Carlos Eduardo Bezerra de Moura

**Affiliations:** 1 Departamento de Morfologia, Universidade Federal do Rio Grande do Norte, Natal, RN, Brasil; 2 Departamento de Ciências Morfológicas, Universidade Estadual de Maringá, Maringá, PR, Brasil; 3 Departamento de Biologia Animal, Universidade Federal de Viçosa, Viçosa MG, Brasil; 4 , Universidade Federal Rural do Semi-Árido, Mossoró, RN, Brasil

**Keywords:** histomorphometry, intertubule, seminiferous tubules, spermatogenesis, Walker carcinoma

## Abstract

Glutamine is often used to treat metabolic changes associated with anorexia-cachexia syndrome in patients with malignant neoplasms. Walker 256 tumor is an excellent model for studying these changes associated with cancer in different organs, including injuries in testicular functions. However, the effects of supplementing glutamine on testicular morphometry in this model have not yet been investigated. Thus, the objective of this study was to evaluate the effect of L-glutamine supplementation on testicular morphometry in rats transplanted with Walker 256 tumor cells. Forty puberty Wistar rats were divided into four groups: control without L-glutamine (C); control supplemented with L-glutamine (CG); inoculated with Walker 256 tumor cells (WT) and inoculated with Walker 256 tumor cells and supplemented with L-glutamine (WTG). The testicles were removed, weighed, fixed in Bouin, and included in paraffin for histomorphometric analysis. Walker 256 tumor caused quantitative changes in the tubular and intertubular compartments and tunica albuginea, with reductions in the percentages of lumen and tunica albuginea, number of Sertoli cells per gram of testis; number of Leydig cells; percentage of blood vessels and connective tissue in intertubule. However, glutamine supplementation prevented part of these changes caused by the tumor, presenting mainly a protective effect on the tunica albuginea and percentage of blood and lymph vessels in the intertubule. These results indicate the potential of L-glutamine was able to recover for testicular dysfunction associated with cancer.

## Introduction

The Walker 256 tumor was discovered by George Walker in 1928 in the region of the mammary gland of a pregnant albino rat. This tumor has become one of the few experimental models available with high rate of proliferation, rapid development, rare inoculation failures, and spontaneous regression (Moraes et al., 2000; Shaughnessy et al., 1991; Silva et al., 2002; Simpkins et al., 1991). These characteristics make the Walker 256 tumor induction a widely used model to test new antitumor substances since resistance to therapies and the high toxicity of chemotherapy and radiotherapy treatments are a major public health challenge (Brito et al., 2007).

One of the mechanisms of action of tumor cells is the ability to use various sources of energy such as glucose, lipids, ketone bodies, and amino acids. For this reason, patients with Walker 256 tumor have hypoglycemia with progressive weight loss (Lira et al., 2012; Martignoni et al., 2003). Tumor cells have low concentration of glutamine, leaving individuals susceptible to the action of amino acid transport blockers. Thus, the use of drugs with functions like glutamine represents an important clinical intervention (Medina et al., 1992).

L-glutamine is a non-essential amino acid which is found most abundantly in blood plasma and regulates the cellular redox balance, oxidative metabolism, apoptosis, cell signaling pathways, and tumor cell proliferation (Cruzat et al., 2009; Matés et al., 2002; Rowbottom et al., 1996). The use of glutamine analogs to combat oxidative stress is feasible since glutamate, a precursor in glutathione biosynthesis, is a nonenzymatic cellular antioxidant in the body. It is known that the increase in oxidative stress is an important factor that causes damages to cellular constituents, since reactive oxygen species may be involved with the development of several pathophysiological processes such as cancer (Cruzat et al., 2007; Pande and Flora, 2002).

Certain tumors directly affect the male reproductive system and radiotherapy and chemotherapy treatments can alter the spermatogenic process and, consequently, compromise fertility (Jones et al., 1994; Suarez-Ibarrola et al., 2018). Experimental models of Walker 256 tumor demonstrated testicular damages such as metabolic changes in Sertoli cells, deficiency of gonadotrophic hormones, changes in vascular perfusion, testicular atrophy, and necrosis (Montemurro, 1960; Paskins-Hurlburt et al., 1982). However, there are still few testicular morphometric studies of animals with Walker 256 tumor as well as studies of L-glutamine actions on tumor injuries in the male gonad. Therefore, the present study aims to evaluate the effects of L-glutamine on tubular and intertubular morphometry of rats with Walker 256 tumor.

## Material and methods

### Animals

This study was approved by the Ethics Committee on Animal Use of the Universidade Estadual de Maringá (protocol nº 099/2012). Forty sexually mature male Wistar rats (*Rattus norvegicus*) at the age of 55 days were obtained from the Central Biotery of the State University of Maringá and kept under controlled illumination (12h light / 12h dark) and temperature (22±2ºC). The animals were housed in polypropylene cages and supplied with water and food *ad libitum.* The animals were randomized into four experimental groups (*n=*10): control without L-glutamine (C); control supplemented with L-glutamine (CG); inoculated with Walker 256 tumor cells (WT) and inoculated with Walker 256 tumor cells and supplemented with L-glutamine (WTG). The animals received daily treatment for 14 days. At the end of the experimental period, animals were fasted for 12 h before being weighed and euthanized under intraperitoneal anesthesia (40 mg/kg body weight thiopental – Abbott Laboratories, Chicago, IL, USA).

### Walker 256 tumor cells culture

Walker 256 tumor cells were maintained in the laboratory with weekly passages by aseptic intraperitoneal injection of 2x10^6^ cells/animal according to Guarnier et al. (2010) method. After seven days of ascitic growth, the peritoneal exudate was removed and subjected to centrifugation. Tumor cells were resuspended in phosphate-buffered saline (PBS) 16.5 mM, pH 7.5, for counting using a Neubauer chamber. The viability of tumor cells was assessed by the Trypan blue exclusion test.

### Walker 256 tumor cells transplantation

Animals carrying Walker 256 tumor were obtained by inoculating a suspension of tumor cells with 8x10^7^ viable cells in 0.5 ml PBS 16.5 mM, pH 7.5, per animal, through injection on the right flank. Control groups (C and CG) were inoculated with 16.5 mM PBS, pH 7.5, at the same site (Guarnier et al., 2010).

### L-Glutamine supplementation

Supplemented animals (CG and WTG groups) received L-glutamine (Deg Importação de Produtos Químicos Ltda, São Paulo, Brazil) incorporated into the standard food (2g glutamine / 100g food). Non-supplemented animals (groups C and WT) received a balanced standard rodent feed (Nuvilab®).

### Histological processing

The testicles were removed, weighed, and fixed in Bouin for 24 hours. Fragments of the testis were subjected to routine histological processing for inclusion in paraffin, sectioned at 3 µm in thickness using a rotatory microtome (RM2255, Leica Microsystems, Heidelberg, Germany), and stained with Hematoxylin and Eosin (HE). Digital images were obtained using microscope (Motic BA410, Motic Causeway Bay, Hong Kong, China) connected to digital camera (Moticam 5.0 MP, Motic Instruments Inc., Richmond, Canada), and analyzed using Image Pro Plus^®^ (Media Cybernetics Inc., Rochvill, USA) software.

### Tubular morphometry

Tubular morphometric analyzes were performed according to the protocols proposed by Braz et al. (2018). The volumetric proportions of seminiferous tubules (tunica propria, seminiferous epithelium and lumen), intertubule and tunica albuginea were determined after counting 2660 intersection points per animal, placed randomly over 10 digital images (266 intersections/points each). The percentage of each component was estimated by dividing the number of obtained points for each component by the total of quantified points per image (266), multiplying this value by 100. The volume of the seminiferous tubule was estimated from the knowledge of its percentage within the testis and the knowledge of the testicular parenchyma weight.

The gonadosomatic index (GSI) represents the investment in the testicles regarding the total body mass and was calculated by dividing the testes weight by body weight and multiplied by 100. The tubulesomatic index (TSI) was calculated in order to quantify the investment in the seminiferous tubules regarding to body mass and was obtained by dividing the tubular volume by body weight and multiplied by 100.

The mean tubular diameter and the seminiferous epithelium height were obtained from the measurement of 20 random circular seminiferous tubules cross section from each animal regardless the tubular stage. The seminiferous tubules length (STL), in meters, was estimated using the formula: STL = STV/ πR^2^, where STV = seminiferous tubule volume, πR^2^ = seminiferous tubule area, R = diameter/2. The seminiferous tubules length per gram of testis (STL/g) was calculated by dividing the STL by testicular weight.

### Cell counting

The estimation of the number of cells was carried out in the seminiferous epithelium at stage 8 of the seminiferous epithelium cycle (SEC), characterized according to the tubular morphology system, in 10 random tubular cross sections per animal. The following cell types were quantified: type A spermatogonia (A), primary spermatocyte at preleptotene-leptotene (PL-L), primary spermatocyte at pachytene (PC), round spermatid (RS) and Sertoli cell (S). Thirty nuclear diameters of each germ cell type and nucleolar diameters of the Sertoli cells were measured for each animal (Russell et al., 1993). The results were corrected according to the thickness section and the nuclear/nucleolar diameters obtained as proposed by Abercrombie (1946) and modified by Amann (1962).

The following indexes were calculated from the corrected numbers of germ and Sertoli cells: mitotic index (PL-L: A), to determine the loss or degeneration that occurred during the spermatogonial mitosis; meiotic index (RS: PC), to determine the efficiency of the meiotic divisions; spermatogenic yield (RS: A), to quantify the efficiency of the spermatogenic process; and the support capacity of Sertoli cells (A + PL-L + PC + RS: S).

The number of Sertoli cells per testis was obtained by multiplying their corrected number by the seminiferous tubules length per testis (in μm) and dividing the result by the section thickness. The obtained results were divided by the testicular weight in order to calculate the number of Sertoli cells per gram of testis. The spermatic reserve of the testis (SRT) was calculated using the formula: SRT = (seminiferous tubule length / cut thickness) × corrected number of round spermatids per cross-section. SRT value was divided by the gonadal weight to obtain the SRT per gram of testis (SRT/g).

### Stage frequency of the seminiferous epithelium cycle (SEC)

The stages of the SEC were characterized by the Tubular Morphology System proposed by Berndston (1977). The relative frequency of the stages was determined after randomly counting 200 seminiferous tubules cross sections per animal. The frequency of each stage was obtained from the average of the counted tubules in each stage multiplied by 100, divided by the number of quantified tubules.

### Intertubular morphometry

Intertubular morphometric analyzes were performed according to the protocols proposed by Silva et al. (2019). The volumetric density of intertubular components (nucleus and cytoplasm of Leydig cell, blood vessels, lymphatic space, and connective tissue) was determined by counting 1000 intersection points, per animal, over intertubular images. The volumetric density of each component in the intertubule was obtained using the following formula: (number of points on the component x 100/1000). The volumetric density of the components in the testicles was obtained using the formula: (% of the intertubule x % of the component in the intertubule / 100). The volume (mL) of each component was calculated using the following formula: (volumetric density of the element in the testes × testicular parenchyma weight)/100. The tunica albuginea weight was subtracted from the testicular weight in order to obtain the testicular parenchyma weight.

The mean diameter of the Leydig cell nucleus was obtained by measuring 30 nuclei per animal, showing characteristic perinuclear chromatin and evident nucleoli. The nuclear (NV, µm^3^) and cytoplasmic (CV, µm^3^) volumes volume of the Leydig cells were obtained using the respective formulas: NV = 4/3πR^3^, where R = nuclear diameter/2; CV = (% of cytoplasm x NV) / % of nucleus. The single Leydig cell volume was calculated by adding the NV and CV.

The number of Leydig cells (NLC) was obtained by dividing the volume occupied by these cells in the testicular parenchyma (µm^3^) by the Leydig cell individual volume (µm^3^). The number of Leydig cells per gram of testis was obtained by dividing the NLC by the total testicular weight. The Leydigosomatic index (LSI, %), which quantifies the energy investment in Leydig cells related to body mass, was estimated by the formula: LSI = total volume of Leydig cell in the testicular parenchyma/BW × 100, where BW = body weight

### Statistical analysis

The data were submitted to analysis of variance (ANOVA), followed by the Student Newman-Keuls post hoc method. The differences were considered significant when p < 0.05. The results were expressed as mean ± standard deviation of the mean.

## Results

### Tubular morphometry

**Figure 1:**
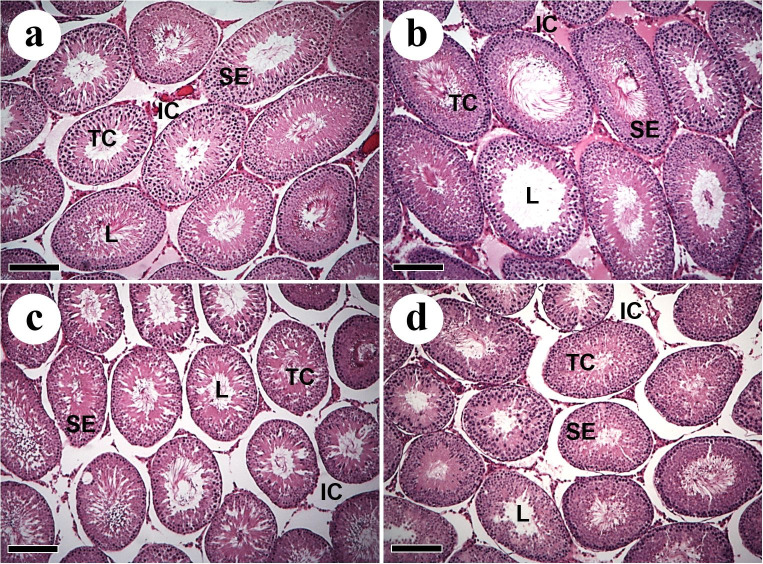
Cross sections of the testicular parenchyma of Wistar rats with Walker 256 tumor and L-glutamine supplementation. (a) Control; (b) Control supplemented with L-glutamine; (c) Walker 256 tumor; (d) Walker 256 tumor supplemented with L-glutamine. TC: tubular compartment; L: lumen; SE: seminiferous epithelium; IC: intertubular compartment. Sections stained in H&E. Scale bars: 15 µm.

**Figure 2:**
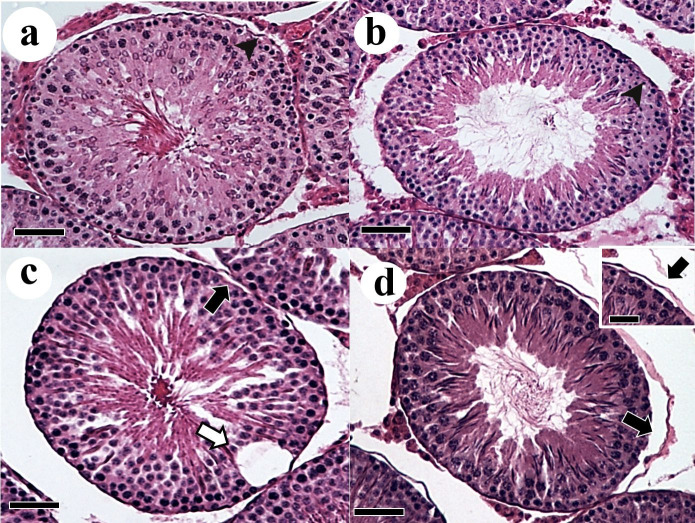
Higher magnification of testicular parenchyma sections of Wistar rats with Walker 256 tumor and L-glutamine supplementation. (a) Control; (b) Control supplemented with L-glutamine; (c) Walker 256 tumor; (d) Walker 256 tumor supplemented with L-glutamine. Degeneration on the seminiferous epithelium (white arrow). Detail in d showing detachment of tunica propria (black arrow). Tunica propria (arrowhead); Sections stained in H&E. Scale bars: 15 µm.

The testicular morphology observed in the experimental groups is shown in Figure 1 and 2. Seminiferous tubules were composed of tunica propria, seminiferous epithelium and lumen. In the WT groups it were observed some areas of degeneration in the seminiferous epithelium (Figure 1 c and 2c) and tunica propria detachment (Figure 1d).

The tumor reduced body weight in animals of WT compared to the control group (C). However, the L-glutamine supplementation significantly increased body weight of WTG compared to WT (p<0.05). There were no significant alterations in gonadal weight and GSI between the experimental groups. However, the percentage of tunica albuginea was significantly lower in WT compared to C, and L-glutamine supplementation was able to raise this parameter in WTG compared to WT (Table 1).

**Table 1:** Biometry and testicular morphometry of Wistar rats with Walker 256 tumor and supplemented with L-glutamine.

**Parameters**	**Experimental groups**			
Body weight (g)	299.60±14.88^Aa^	295.40±23.58^Ca^	257.60±15.85^Bc^	284.40±10.43^Cd^
Gonadal weight (g)	2.87±0.25	2.69±0.36	2.71±0.23	2.76±0.11
Gonadosomatic index (GSI, %)	0.96 ±0.05	0.91±0.09	1.05±0.07	0.97±0.04
Tunica Albuginea (%)	1.44±0.40^Aa^	1.33±0.25^Ca^	0.66±0.12^Bc^	1.02±0.24^Cd^
Seminiferous tubules (%)	74.64±6.42	73.73±4.22	70.42±2.10	71.28±1.42
Tunica propria (%)	3.66±0.74	4.12±0.72	4.44±0.34	5.11±1.35
Lumen (%)	29.47±2.84^Aa^	25.74±1.76^Cb^	24.30±1.74^Bc^	24.21±1.41^Cc^
Seminiferous epithelium (%)	41.51±4.74	43.87±4.21	41.68±1.24	41.96±0.86
Intertubule (%)	25.36±6.42	26.27±4.22	29.58±2.10	28.72±1.42
Seminiferous tubules volume (mL)	2.11±0.21	1.97±0.34	1.90±0.12	1.95±0.09
Tubulesomatic index (TSI, %)	0.70±0.06	0.66±0.10	0.74±0.05	0.69±0.04
Tubular diameter (µm)	128.55±3.10^Aa^	125.54±4.10^Ca^	121.23±5.31^Ac^	116.14±4.08^Dc^
Epithelium height (µm)	26.72±0.33	28.46±1.43	26.18±2.91	27.36±2.90
Seminiferous tubules length (m)	162.74±20.05	160.51±38.49	164.67±13.41	184.39±9.97
Seminiferous tubules length per gram of testis (m/g)	56.97±7.56	59.09±6.86	60.97±6.23	66.79±4.10

C: Control; CG: Control supplemented with L-glutamine, WT: Walker 256 tumor; WTG: Walker 256 tumor supplemented with L-glutamine. Averages with different pairs of capital letters on the same line (A-B; C-D) show comparisons between control (C x WT) and supplemented groups (CG x WTG), respectively. Averages with different pairs of lower case letters on the same line (a-b; c-d) show comparisons between control (C x CG) and Walker Tumor groups (WT x WTG), respectively. Data are expressed as mean ± standard deviation of the mean (p≤0.05).

Considering all the experimental groups, the testicular parenchyma of animals in this study was composed by 72.51% of seminiferous tubules and 27.48% of intertubule. The tumor reduced lumen percentage in WT compared to C, and L-glutamine supplementation was not able to raise this parameter in WTG. In addition, in the supplemented groups, the tubular diameter decreased in WTG compared to CG (Table 1). The other tubular morphometric parameters evaluated did not show statistical variation between the experimental groups.

### Cell counting

The tumor reduced the number of Sertoli cells in WT compared to C, and L-glutamine supplementation had a protective effect on these cells in the WTG group compared to WT. Primary spermatocytes in preleptotene-leptotene increased in WT compared to C, as well as in CG compared to C. However, L-glutamine supplementation did not increase the proliferation of these cells in animals with the tumor. Similarly, the support capacity of Sertoli cells was greater in WT compared to the control group (C). However, L-glutamine supplementation reduced this capacity in WTG compared to WT. The number of Sertoli cells per gram of testis was lower in WT compared to the control group (C). The L-glutamine supplementation had a considerable protective effect on the total number of these cells per gram of testis in WTG compared to CG and WT (Table 2).

**Table 2:** Corrected number of cells at stage 8 of the seminiferous epithelium cycle (SEC), spermatogenic indexes, Sertoli cells number and spermatic reserve of the testis of Wistar rats with Walker 256 tumor and supplemented with L-glutamine.

**Parameters**	**Experimental groups**			
Sertoli cell	16.08±1.48^Aa^	17.32±0.90^Ca^	13.02±0.91^Bc^	17.36±1.45^Cd^
Type A spermatogonia	4.60±0.30	3.88±0.39	4.46±0.34	5.58±1.43
Primary spermatocyte at preleptotene-leptotene	26.72±3.82^Aa^	35.46±2.49^Cb^	35.06±1.59^Bc^	37.50±2.32^Cc^
Primary spermatocyte at pachytene	50.50±1.35	51.14±2.01	49.00±6.61	44.20±5.63
Round spermatid	189.38±7.07	191.20±12.84	179.74±15.74	173.14±18.57
Mitotic index	5.85±1.04	9.16±0.70	7.88±0.46	7.04±1.62
Meiotic index	3.75±0.12	3.74±0.27	3.69±0.25	3.96±0.55
Spermatogenic yield	41.37±3.84	49.68±6.67	40.67±6.47	32.74±8.93
Sertoli cell support capacity	16.95±1.67^Aa^	16.30±2.20^Ca^	20.67±2.03^Bc^	15.06±1.44^Cd^
Sertoli cell number per testis (10^11^)	8.64±0.10	7.53±0.85	5.67±0.79	10.10±1.65
Sertoli cell number per gram of testis (10^11^)	3.04±0.55^Aa^	2.80±0.16^Ca^	2.10±0.33^Bc^	3.67±0.72^Dd^
Spermatic reserve of the testis (10^7^)	1.01±0.03	1.10±0.09	0.78±0.09	1.00±0.14
Spermatic reserve per gram of testis (10^6^)	3.56±0.44	4.11±0.43	2.88±0.37	3.64±0.58

C: Control; CG: Control supplemented with L-glutamine, WT: Walker 256 tumor; WTG: Walker 256 tumor supplemented with L-glutamine. Averages with different pairs of capital letters on the same line (A-B; C-D) show comparisons between control (C x WT) and supplemented groups (CG x WTG), respectively. Averages with different pairs of lower case letters on the same line (a-b; c-d) show comparisons between control (C x CG) and Walker Tumor groups (WT x WTG), respectively. Data are expressed as mean ± standard deviation of the mean (p≤0.05).

### Stage frequency of the seminiferous epithelium cycle (SEC)

**Figure 3:**
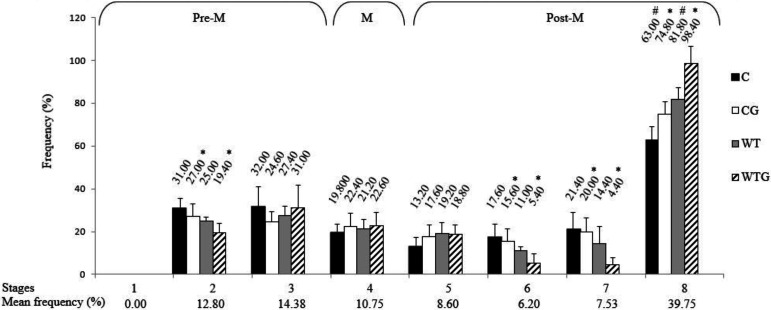
Frequency of the stages of the seminiferous epithelium cycle of Wistar rats with Walker 256 tumor and with L-glutamine supplementation. Pre-M: pre-meiotic phase; M: meiotic phase; Post-M: post-meiotic phase. C: Control; CG: Control supplemented with L-glutamine; WT: Walker 256 tumor; WTG: Walker 256 tumor supplemented with L-glutamine. Symbols indicate differences between groups, within each stage (*: CG x WTG comparison; #: C x WT comparison).

The animals did not show stage 1 of the SEC. Stage 8 was the most frequent and stage 6 the least frequent stage in all experimental groups. The frequencies were grouped and divided into the pre-meiotic (stages 1 to 3), meiotic (stage 4), and post-meiotic (stages 5 to 8) phases, which represented 27.18%, 10.75%, and 62.08% of the SEC, respectively (Figure 3).

Comparing the frequency of stages between the experimental groups, it was observed variation in stages 2, 6, 7, and 8. Stages 2, 6, and 7 were more frequent in CG compared to WTG. Stage 8 was always more frequent in animals with the tumor, whether in groups supplemented with L-glutamine or not (Figure 3).

### Intertubular morphometry

**Figure 4:**
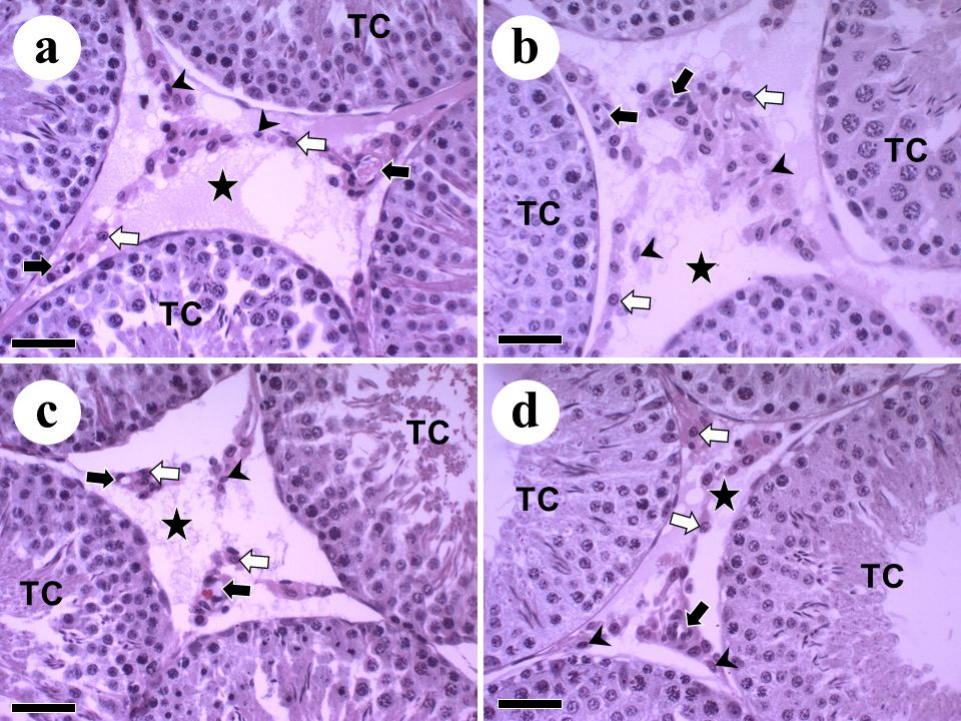
Intertubular compartment of Wistar rats with Walker 256 tumor and with L-glutamine supplementation. (a) Control; (b) Control supplemented with L-glutamine; (c) Walker 256 tumor; (d) Walker 256 tumor supplemented with L-glutamine. Leydig cell (white arrow); blood vessels (black arrow); lymphatic vessel (star); Connective tissue (arrowhead). TC: tubular compartment; Sections stained in H&E. Scale bars: 40 μm.

The intertubular compartment was predominantly constituted by Leydig cells, followed by lymphatic vessels, blood vessels and connective tissue, with no apparent morphological changes (Figure 4). The tumor (WT) reduced the percentage of blood vessels in the intertubule compared to C. L-glutamine supplementation also reduced the percentage of blood vessels (C versus GC). However, when comparing the WT and WTG groups, it was observed that supplementation had a preventive effect in reducing this percentage of blood vessels. In relation to lymphatic vessels, the tumor (WT) increased the percentage of these vessels in the intertubule in relation to C, occupying the largest proportion within the intertubule. The treatment with L-glutamine (WTG) reduced this parameter reaching values similar to those of the control group (C). Although glutamine stimulates lymphangiogenesis in the GC group, this stimulation was not significant and therefore indicates the beneficial effect of glutamine in the WTG group. The tumor reduced the percentage of connective tissue in both the intertubule and the testicles, as well as its volume in the testicles in relation to the control (Table 3). The tumor reduced the percentage of connective tissue on both intertubule and testes, as well as its volume in the testes compared to the control (Table 3).

**Table 3:** Intertubular morphometry of Wistar rats with Walker 256 tumor and supplemented with L-glutamine.

**Parameters**	**Experimental groups**			
Intertubule volume (mL)	0.72±0.20	0.69±0.09	0.79±0.11	0.78±0.050
Volumetric density in the intertubule (%)				
Leydig cell	17.66±3.14	17.00±3.61	13.62±3.30	16.96±3.77
Blood vessel	7.30±3.74^Aa^	2.60±2.67^Cb^	2.84±1.94^Bc^	3.12±0.92^Cd^
Lymphatic vessel	70.28±3.23^Aa^	78.78±3.93^Ca^	81.56±4.75^Bc^	78.06±3.57 ^Cc^
Connective tissue	4.76±1.58^Aa^	1.62±0.76^Cb^	1.98±0.24^Bc^	1.86±0.51 ^Cc^
Volumetric density in the testes (%)				
Leydig cell	4.35±0.72	4.38±0.67	4.01±0.93	4.88±1.20
Blood vessel	1.90±1.06	0.68±0.72	0.81±0.51	0.90±0.29
Lymphatic vessel	17.89±4.97	20.79±4.08	24.18±2.84	22.40±1.15
Connective tissue	1.22±0.55^Aa^	0.42±0.20^Cb^	0.59±0.10^Bc^	0.53±0.14^Cc^
Volume in the testes (mL)				
Leydig cell	0.1231±0.02	0.1163±0.02	0.1073±0.02	0.1344±0.03
Blood vessel	0.0557±0.03	0.0182±0.01	0.0212±0.01	0.0246±0.008
Lymphatic vessel	0.5081±0.15	0.5448±0.08	0.6556±0.12	0.6123±0.02
Connective tissue	0.0349±0.01^Aa^	0.0113±0.005^Cb^	0.0159±0.003^Bc^	0.0145±0.003 ^Cc^

C: Control; CG: Control supplemented with L-glutamine, WT: Walker 256 tumor; WTG: Walker 256 tumor supplemented with L-glutamine. Averages with different pairs of capital letters on the same line (A-B; C-D) show comparisons between control (C x WT) and supplemented groups (CG x WTG), respectively. Averages with different pairs of lower case letters on the same line (a-b; c-d) show comparisons between control (C x CG) and Walker Tumor groups (WT x WTG), respectively. Data are expressed as mean ± standard deviation of the mean (p≤0.05).

Leydig cells morphometry is shown in Table 4. The tumor reduced the number of Leydig cells in the testis and per gram of testis compared to the control group, and L-glutamine supplementation had no significant effect on preventing these changes.

**Table 4:** Leydig cells morphometry of Wistar rats with Walker 256 tumor and supplemented with L-glutamine.

**Parameters**	**Experimental groups**			
Nuclear diameter (µm)	6.97±0.12	7.18±0.38	6.83±0.28	7.11±0.14
Nuclear percentage (%)	39.92±6.20	36.75±6.16	33.52±4.61	32.91±3.82
Nuclear volume (μm^3^)	177.73±9.10	195.51±31.07	167.99±20.77	188.59±11.15
Cytoplasmic percentage (%)	60.08±6.20	63.25±6.16	66.48±4.61	67.09±3.82
Cytoplasmic volume (μm^3^)	276.18±72.75	355.65±133.15	340.79±86.13	393.04±90.54
Leydic cell volume (μm^3^)	453.91±72.77	551.15±159.37	508.78±98.91	581.63±97.20
Number of Leydig cells in the testis (10^8^)	2.71±0.29^Aa^	2,19±0.53 ^Ca^	1.96±0.23^Bc^	2.28±0.38 ^Cc^
Number of Leydig cells per gram of testis (10^7^)	9.45±0.41^Aa^	8.12±0.12 ^Ca^	7.27±0.11^Bc^	8.27±0.12 ^Cc^
Leydigosomatic index (LSI, %)	0.041±0.007	0.039±0.005	0.042±0.010	0.047±0.012

C: Control; CG: Control supplemented with L-glutamine, WT: Walker 256 tumor; WTG: Walker 256 tumor supplemented with L-glutamine. Averages with different pairs of capital letters on the same line (A-B; C-D) show comparisons between control (C x WT) and supplemented groups (CG x WTG), respectively. Averages with different pairs of lower case letters on the same line (a-b; c-d) show comparisons between control (C x CG) and Walker Tumor groups (WT x WTG), respectively. Data are expressed as mean ± standard deviation of the mean (p≤0.05).

## Discussion

There has been an increasing number of men diagnosed with cancer, especially testicular cancer, which can trigger infertility, impact their quality of life and cause prolonged toxicity due to drugs employed in the treatment (Chen et al., 2019; Punab et al., 2017). However, it has been shown that this type of cancer is one of the most curable malignancies, which reinforces the need for studies to understand the progression of the disease in search for early therapy (Adra and Einhorn, 2017; Howlader et al., 2019; Smith et al., 2018). The search for alternative therapies can assist therapeutic intervention of male infertility, transient or permanent, resulting from chemotherapy and/or radiotherapy. Therefore, it is relevant to understand the spermatogenesis in a model of neoplasia, such as Walker 256 tumor, and its effects on testicle morphometry as well as the antioxidant effect of L-glutamine, which is still little known.

### Biometry and tubular morphometric analysis

Walker 256 tumor reduced body weight of the animals in this study as well as some testicular morphometric parameters. Reduction in body weight is commonly seen in this tumor (Gomes-Marcondes et al., 2003), which is probably due to increased catabolic metabolism promoted by the tumor (Lousan and Toledo, 2012). However, glutamine supplementation recovered body weight probably because this antioxidant increased intramuscular and plasma glutamine, and consequently, protein synthesis (Cella et al., 2020; Yoshida et al., 2001). These results agree with researches that previously demonstrated the positive effects of glutamine on cancer treatment (Boligon and Huth, 2011; Santos and Novaes, 2011; Savarese et al., 2003).

The testicles are covered by a connective tissue capsule, called tunica albuginea, which surrounds the entire gonad (Johnson et al., 1981). In addition, the albuginea is related to the contractile properties of testicular spermiation, control of blood flow and maintenance of interstitial pressure due to the presence of smooth muscle cells (Banks et al., 2006; Davis and Langford, 1969; Middendorff et al., 2002; Setchell and Breed, 2006). The reduced percentage of tunica albuginea in animals with Walker 256 tumor may be related to the reduction in the testis contractile capacity which is required for spermiation (Banks et al., 2006), since it has been demonstrated a decrease in muscle contraction capacity in rats with this tumor (Gomes-Marcondes et al., 2003). In this same study, food supplementation with the amino acid leucine softened these effects, as observed in the present work regarding the increase in the percentage of tunica albuginea after supplementation with L-glutamine.

Although the testicular weight and gonadosomatic index did not differ significantly in treatments, the tumor significantly reduced the percentage of lumen in the testicular parenchyma. This may be a reflect from an attempt of the seminiferous epithelium to maintain the spermatogenesis face the tumor, which was reinforced by the increase in the population of primary spermatocytes in preleptotene/leptotene when comparing the C and WT groups. This can be hypothesized since these spermatocytes are the first meiotic cells to be formed, and then will generate all the others germ cells (Lara et al., 2018).

There was also a reduction in the diameter of seminiferous tubules in WTG compared to CG. However, despite tubular diameter is directly related to spermatogenic activity (Sinha-Hikim et al., 1988; Predes et al., 2010), this alteration wasn’t followed by a reduction in the parameters evaluated for the seminiferous epithelium, which could reflect more precisely a spermatogenic disturbance. So, this reduction isolated is not enough to infer a significative damage to the spermatogenesis.

### Cell counting and stages of the seminiferous epithelium cycle (SEC)

The quantification of cell population in stage 8 of the SEC revealed that the tumor had an impact only on primary spermatocytes in preleptotene-leptotene and Sertoli cells without affecting spermatogenic indexes. Although the tumor reduced the population of Sertoli cells and their number per gram of testis, it was observed an attempt to recover the seminiferous epithelium by the increase in the number of primary spermatocytes in preleptotene-leptotene and by the increase in the support capacity of Sertoli cells in all groups. This increase in PL / L spermatocytes may be related to interaction of Sertoli cells with cytokines or perhaps testosterone. Mitotic factors some interference whereas after the last spermatogonial division, there is only differentiation in primary spermatocytes to initiate meiosis and this is where this increase is apparent (Lara et al, 2018).

Considering that Sertoli cells are responsible for the nutritional and structural support necessary for spermatogenesis (Griswold and McLean, 2005), the increase in the support capacity of these cells may be related to the attempt on minimizing the damage to seminiferous epithelium caused by the tumor. The reduction in the number of Sertoli cells can also be related to the reduction in the transport of fluids through seminiferous tubules since these fluids are also produced by the Sertoli cells (Ghosh et al., 1992; Sharpe, 1989). However, L-glutamine supplementation showed considerable protective effect for Sertoli cells comparing CG and WTG. There was a significant increase in the number of Sertoli cells per gram of testis in WTG compared to WT group. This increase may be related to the tumor affinity for glutamine, as previously demonstrated in Walker 256 tumor cell culture (Neuman and Mccoy, 1956). Meanwhile, the reduction in the Sertoli cell support capacity observed in WTG compared to WT group may be related to the increase in the number of these cells per gram of testis.

The determination of phases and frequency of the seminiferous epithelim cycle (SEC) are very important in quantitative studies to evaluate spermatogenesis since they allow to detect possible cellular degenerations (Berndston, 1977). In animals that received supplementation with L-glutamine, the tumor reduced the frequency of stages 2, 6 and 7, whereas the opposite was observed for stage 8. Although the frequency of the SEC stages is considered relatively constant within the same species, it is known that variations can occur in relation to exogenous and pathological factors, which indicates deleterious effects on spermatogenesis (Bartlett et al., 1990; Rosiepen et al., 1995; França and Russell, 1998). In fact, on this study we observed harmful effects of Walker's tumor on the normal progression of spermatogenesis, whereas supplementation with L-glutamine seems to have had a protective effect against the normal progression of stages 2, 6 and 7. The increase in stage 8 frequency in WTG group compared to CG may be related to the apparent attempt to recover the seminiferous epithelium in response to tumor, similar to that observed for the number of Sertoli cells per gram of testis. The increase in the frequency of this stage in WT group compared to C was similar to the findings of greater support capacity of Sertoli cells as a response to tumors.

The division of the SEC in pre-meiotic, meiotic and post-meiotic phases allows to compare the duration of these phases between different treatments (França and Russell, 1998; Morais et al., 2013). The duration of meiotic phase was similar to that reported in the literature. However, while the pre-meiotic is usually the longest phase, we observed a longer duration of post-meiotic phase, which was due to the fact that stage 1 was not observed.

Russell et al. (1983) describe the lack of synchronization cycle as the cause for this finding, suggesting that there may have been an acceleration in the development of germ cells which reflected in the SEC stages observed here. The higher frequency found for the post-meiotic phase (68.08%) in this study may also be associated with the puberty animals, which were in their reproductive activity period (Russell et al., 1993). In addition, acceleration of the post-meiotic phase was demonstrated in the testicles of mice exposed to high temperatures, a harmful exposure to spermatogenesis (Meistrich et al., 1973). Thus, the longer duration of this phase in the present study may also be associated with the deleterious effects of Walker's tumor on spermatogenesis.

### Intertubular morphometry

The intertubular or androgenic compartment presented similar morphology to that found in mammals which is composed of Leydig cells, blood vessels, lymphatic vessels and connective tissue (França and Russell, 1998). This compartment resembles the pattern of the type I described by Fawcett et al. (Fawcett et al., 1973), in which a small amount of Leydig cells and blood vessels are separated from the seminiferous tubules by a very developed lymphatic space. This pattern implies an increase in the ability to maintain androgen concentrations, as well as the testicular ability to eliminate metabolites.

Previous studies reported reduction in the testicular blood flow and concentration of gonadotrophic hormones in rats with Walker tumor related to hormonal imbalance, metabolic and nutritional changes (Montemurro, 1960; Paskins-Hurlburt et al., 1982). We demonstrated that Walker 256 tumor reduced the percentage of blood vessels in the intertubule which can compromise testicular steroidogenesis with consequences on hormonal balance and sperm production, since it was observed depletion in spermatogenesis in rats induced with different testicular ischemia levels (Bergh et al., 2001). However, supplementation with L-glutamine increased the percentage of blood vessels in WTG group compared to WT, suggesting prevention of cancer-related metabolic changes (Montrose and Galluzzi, 2019), as well as the ability of this amino acid to modulate testicular angiogenesis as observed in other tissues (Goswami et al., 2016; Teuwen et al., 2019). Olaniyi et al. (2020) demonstrated an antioxidant effect of glutamine supplementation mediated by the synthesis of glucose-6-phosphate dehydrogenase (G6PD) and nitric oxide with improved blood flow and perfusion in the testicles of rats with testicular dysfunction induced by cadmium chloride. The authors also demonstrated an increase in production of gonadotropins and an improvement in the semen quality of these animals.

The observed reduction in the percentage of blood vessels and connective tissue occurred as a result of the increase in the percentage of lymphatic vessels in animals with Walker 256 tumor compared to the control. The fact that lymphatic vessels promote recirculation of fluids from blood vessels to maintenance of tissue homeostasis but also are routes for cancer metastasis (Hirai et al., 2012) may explain its increase in animals with Walker tumor. Testicular cancer is one of the most common malignancies in young men, and can easily metastasize to the retroperitoneal lymph nodes from the testicular lymphatic vessels (Hirai et al., 2013).

Although the proportions and volume of Leydig cells have not been altered in animals with Walker 256 tumor, the number of these cells in the testis and per gram of testis was lower. This reduction may be associated with an interference in testicular androgenic capacity, since it was shown that patients with non-androgenic cancers showed reduction in serum testosterone levels, which was always linked to the individuals' metabolic status and hypogonadism (Fleishman et al., 2010). The L-glutamine supplementation did not increase the number of Leydig cells suggesting that this amino acid was not able to reduce a possible hormonal stress usually found in tumor situations, as observed in studies with testicular cancer and testicular toxicity, as well as hormonal disorders in organs with endocrine activity, such as prostate and others (Calderón Guzmán et al., 2011; Olaniyi et al., 2020; Rotovnik Kozjek et al., 2017)

## Conclusions

Walker 256 tumor caused quantitative changes in the tubular and intertubular compartments with reductions in the percentages of lumen and tunica albuginea, counting of Sertoli cells, percentage of blood and connective tissue, and number of Leydig cells. Thus, in the presented experimental conditions, we concluded that L-glutamine was able to recover the testicular damages caused by the tumor, such as percentage of tunica albuginea, number of Sertoli cells per gram of testis and the percentage of blood and lymph vessels in the intertubule.
